# 

**Published:** 1886-09

**Authors:** 


Whole Number, 320 SEPTEMBER, 1886. Vol. LIII. Number 3.

LIBRARY
INDEXE[illegible]
S.G.O.

THE

### CHICAGO MEDICAL JOURNAL AND EXAMINER

EDITORS:

S. J. JONES, M.D., LL.D.

W. W. JAGGARD, A.M., M.D. HAROLD N. MOYER, M.D.

PUBLISHED MONTHLY BY

THE CLARK & LONGLEY CO.,

Under direction of

THE CHICAGO MEDICAL PRESS ASSOCIATION,

308-316 Dearborn Street.

